# Cellular Mechanisms of Angiogenesis in Neonatal Rat Models of Retinal Neurodegeneration

**DOI:** 10.3390/ijms20194759

**Published:** 2019-09-25

**Authors:** Daiki Asano, Masaki Hokazono, Shogo Hirano, Akane Morita, Tsutomu Nakahara

**Affiliations:** Department of Molecular Pharmacology, Kitasato University School of Pharmaceutical Sciences, 5-9-1 Shirokane, Minato-ku, Tokyo 108-8641, Japan; asanod@pharm.kitasato-u.ac.jp (D.A.); pp12204@st.kitasato-u.ac.jp (M.H.); pl15038@st.kitasato-u.ac.jp (S.H.); moritaa@pharm.kitasato-u.ac.jp (A.M.)

**Keywords:** endothelial cell, excitotoxicity, retinal neuronal cell, retinal blood vessels, retinal glial cell

## Abstract

Νeuronal and glial cells play an important role in the development of vasculature in the retina. In this study, we investigated whether re-vascularization occurs in retinal neurodegenerative injury models. To induce retinal injury, *N*-methyl-D-aspartic acid (NMDA, 200 nmol) or kainic acid (KA, 20 nmol) was injected into the vitreous chamber of the eye on postnatal day (P)7. Morphological changes in retinal neurons and vasculature were assessed on P14, P21, and P35. Prevention of vascular growth and regression of some capillaries were observed on P14 in retinas of NMDA- and KA-treated eyes. However, vascular growth and re-vascularization started on P21, and the retinal vascular network was established by P35 in retinas with neurodegenerative injuries. The re-vascularization was suppressed by a two-day treatment with KRN633, an inhibitor of VEGF receptor tyrosine kinase, on P21 and P22. Astrocytes and Müller cells expressed vascular endothelial growth factor (VEGF), and the distribution pattern of VEGF was almost the same between the control and the NMDA-induced retinal neurodegenerative injury model, except for the difference in the thickness of the inner retinal layer. During re-vascularization, angiogenic sprouts from pre-existing blood vessels were present along the network of fibronectins formed by astrocytes. These results suggest that glial cells contribute to angiogenesis in neonatal rat models of retinal neurodegeneration.

## 1. Introduction

Glutamate is the main excitatory neurotransmitter in the retina. Glutamate receptors can be divided into two groups. Ionotropic glutamate receptors are ligand-gated ion channels that respond to *N*-methyl-D-aspartic acid (NMDA), alpha-amino-3-hydroxy-5-methyl-4-isoxazolepropionic acid (AMPA), or kainic acid (KA), while metabotropic glutamate receptors function through second messenger systems [1,2]. Overstimulation of NMDA receptors or KA receptors causes significant neuronal cell death in rodent experimental models [3,4,5]. On the other hand, overstimulation of AMPA receptors fails to impact most of neuronal cells, except for choline acetyltransferase (ChAT)-positive cells [6]. Therefore, NMDA and KA have been used for inducing extensive retinal injuries to study the mechanisms of neurodegenerative processes [3,4,5,7].

The mechanisms of glutamate-induced retinal injury involve excessive Ca^2+^ influx and subsequent activation of Ca^2+^-dependent events, leading to the death of neuronal cells [8,9]. In addition to these direct mechanisms for neurotoxicity, indirect mechanisms such as upregulation of pro-inflammatory cytokines, recruitment of leukocytes into the retina, and impairment of retinal circulation may also contribute to neuronal cell death [10,11,12,13,14,15].

Recent evidence suggests that the neuronal–glial–vascular interaction in the retina is important for maintaining retinal homeostasis [16,17]. In addition, there is evidence that the retinal vascular network is not established in mice deficient in retinal ganglion cells (RGCs) [18], and that regression of capillaries and delay in vascular development occur in neonatal rat retina following neuronal cell loss induced by intravitreal injection of NMDA [19]. Thus, retinal neuronal cells play a critical role in the development and maintenance of retinal blood vessels.

Retinal glial cells as well as neuronal cells are also critical for maintaining retinal vascular homeostasis [20,21,22]. Reactive gliosis in both retinal astrocytes and Müller cells occurs in response to glutamate-induced retinal injury [5,7,23]. Therefore, we hypothesized that glial cells contribute to angiogenesis following neurodegenerative injuries. To test this hypothesis, we examined the morphological changes of blood vessels and astrocytes in the retinas of neonatal rats intravitreally injected with NMDA or KA. We also examined the distribution of fibronectin, which provides a guidance cue for migration of endothelial cells [24,25,26], and the role of vascular endothelial growth factor (VEGF) in angiogenesis in retinal neurodegenerative injury models.

## 2. Results

**Figure 1:**
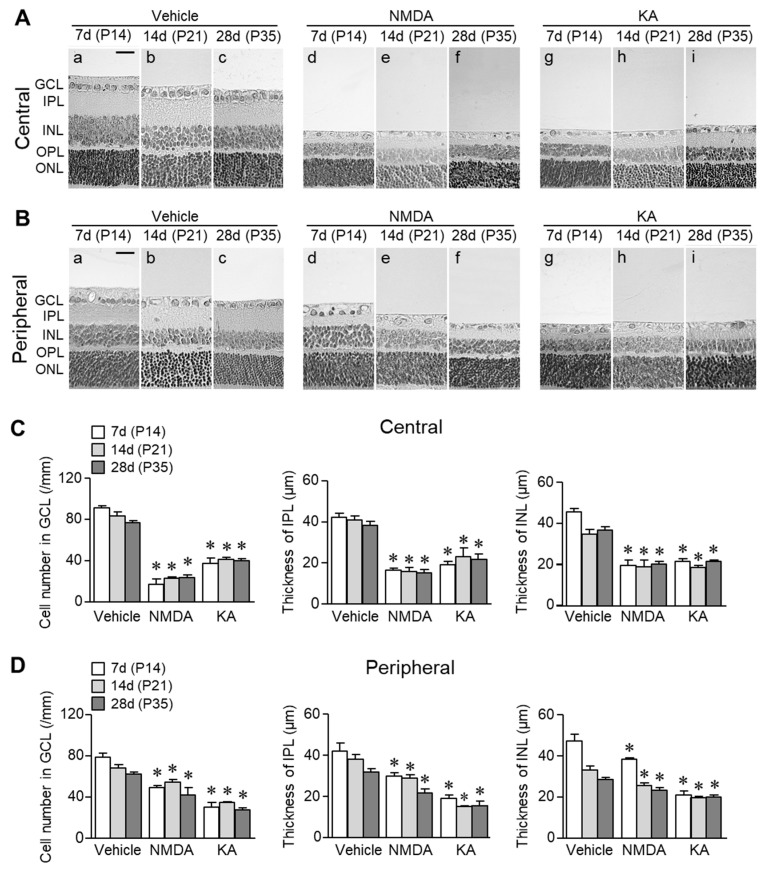
Changes in overall retinal morphology in rats intravitreally injected with NMDA or KA. (**A**,**B**) Representative photomicrographs of retinal cross-sections stained with hematoxylin and eosin obtained 7 (P14), 14 (P21), and 28 days (P35) following intravitreal injection of vehicle, NMDA (200 nmol) or KA (20 nmol) on P7. Scale bar = 50 μm in panel a (applies to b–i). (**C**,**D**) Quantification of the number of cells in GCL and the thicknesses of IPL and INL in central (**C**) and peripheral (**D**) retinal areas. The number of cells in GCL was expressed per length of retina (in millimetres). * *p* < 0.05 vs. the corresponding age-matched control value (vehicle). *n* = 4–5.

Figure 1A,B show representative photomicrographs of retinal cross-sections stained with hematoxylin and eosin obtained 7 (on P14), 14 (on P21), and 28 days (on P35) after intravitreal injection of vehicle, NMDA (200 nmol) or KA (20 nmol) on P7. In vehicle-treated eyes, the number of cells in GCL and the thicknesses of IPL and INL decreased in central (Figure 1A) and peripheral (Figure 1B) retinal areas. Thus, the developmental changes occurred from P14 to P35. In NMDA-treated eyes, a further reduction in the cell number in GCL and the thicknesses of IPL and INL was observed in both central (Figure 1Ad–f) and peripheral (Figure 1Bd–f) retinas. The effects of NMDA on neuronal cells were larger in the central area than in the peripheral area (Figure 1C,D). Intravitreal injection of KA also reduced the cell number in GCL and the thicknesses of IPL and INL (Figure 1Ag–i and Bg–i). However, the degree of neuronal degeneration was almost the same between central (Figure 1Ag–i and C) and peripheral areas (Figure 1Bg–i and D). There was no visible change in OPL and ONL in the NMDA- or KA-treated eyes (Figure 1A,B).

**Figure 2:**
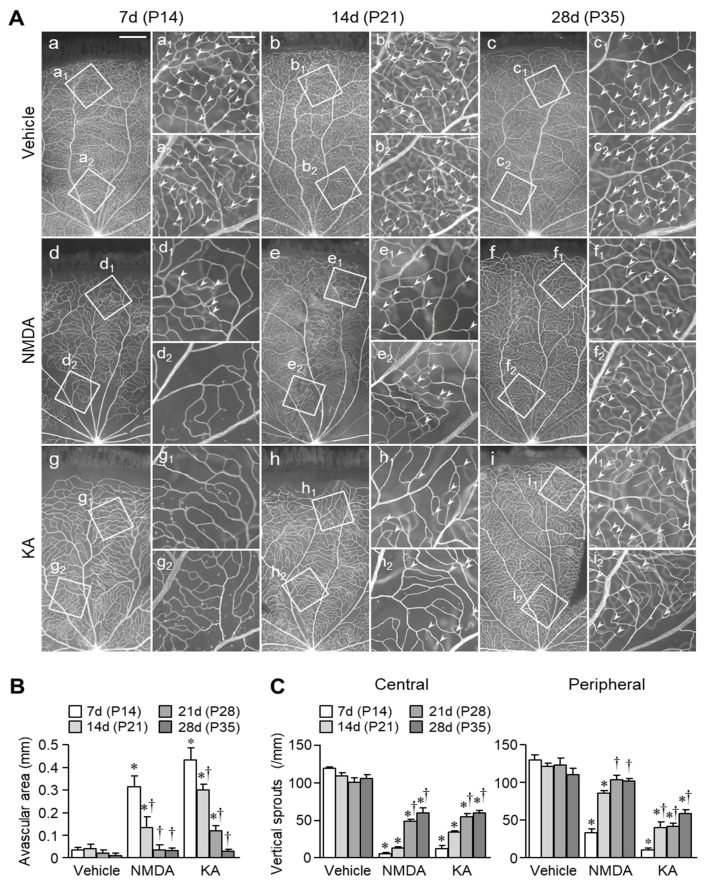
Changes in retinal vasculature in rats intravitreally injected with NMDA or KA. (**A**) Fluorescence photomicrographs of retinal whole-mounts stained with an endothelial cell marker (RECA) obtained 7 (P14), 14 (P21), and 28 days (P35) following intravitreal injection of vehicle, NMDA (200 nmol) or KA (20 nmol) on P7. Higher-magnification images of the insets are shown in the panels Aa_1_–i_1_ and Aa_2_–i_2_. Arrowheads indicate vertical sprouts. Scale bars = 500 μm in a (applies to b–i) and 150 μm in a_1_ (applies to b_1_–i_1_ and a_2_–i_2_). (**B**,**C**) Quantification of the distance from the developing edge of the vascular bed to the peripheral edge of the retina (**B**) and the number of vertical sprouts in central and peripheral retinal areas (**C**). * *p* < 0.05 vs. the corresponding age-matched control value (vehicle). ^†^ *p* < 0.05 vs. the corresponding seven days after the injection. *n* = 4–5.

Representative fluorescence photomicrographs of retinal whole-mounts stained with a marker of endothelial cells are shown in Figure 2. The vascular bed covered the entire retinal surface seven days (on P14) after intravitreal injection of vehicle, but not of NMDA or KA (Figure 2Aa, Ad, and Ag). The size of peripheral avascular area in KA-treated eyes was larger than that in NMDA-treated ones (Figure 2B). However, the vascular bed reached the peripheral edge of retina in both NMDA- and KA-treated eyes 28 days (on P35) after the injection (Figure 2Af and Ai). Many vertical sprouts were observed in central (Figure 2Aa_2_–c_2_) and peripheral (Figure 2Aa_1_–c_1_) retinal areas in vehicle-treated eyes. The number of vertical sprouts decreased seven days (on P14) after the injection of NMDA (Figure 2Ad_1_, Ad_2_, and C), but it increased at 28 days (on P35) (Figure 2Af_1_, Af_2_, and C). The degree of reduction in vertical sprouts was larger in the central area than in the peripheral area (Figure 2C). Similarly, a transient reduction in vertical sprouts was observed in the KA-treated eyes (Figure 2Ag_1_–i_1_, Ag_2_–i_2_, and C). However, no significant difference was found between the central and peripheral retinal areas of KA-treated eyes (Figure 2C). In central retina, capillary vessels regressed seven days (on P14) after intravitreal injection of NMDA or KA (Figure 2Ad and Ag). In NMDA-treated eyes, capillary regression in the central retina was more severe than in the peripheral retina (Figure 2Ad_1_ and Ad_2_), but there was no significant difference between the central and peripheral areas in the KA-treated eyes (Figure 2Ag_1_ and Ag_2_). In both NMDA- and KA-treated eyes, re-vascularization was observed on P21 and later (Figure 2Ad–i, B and C).

**Figure 3:**
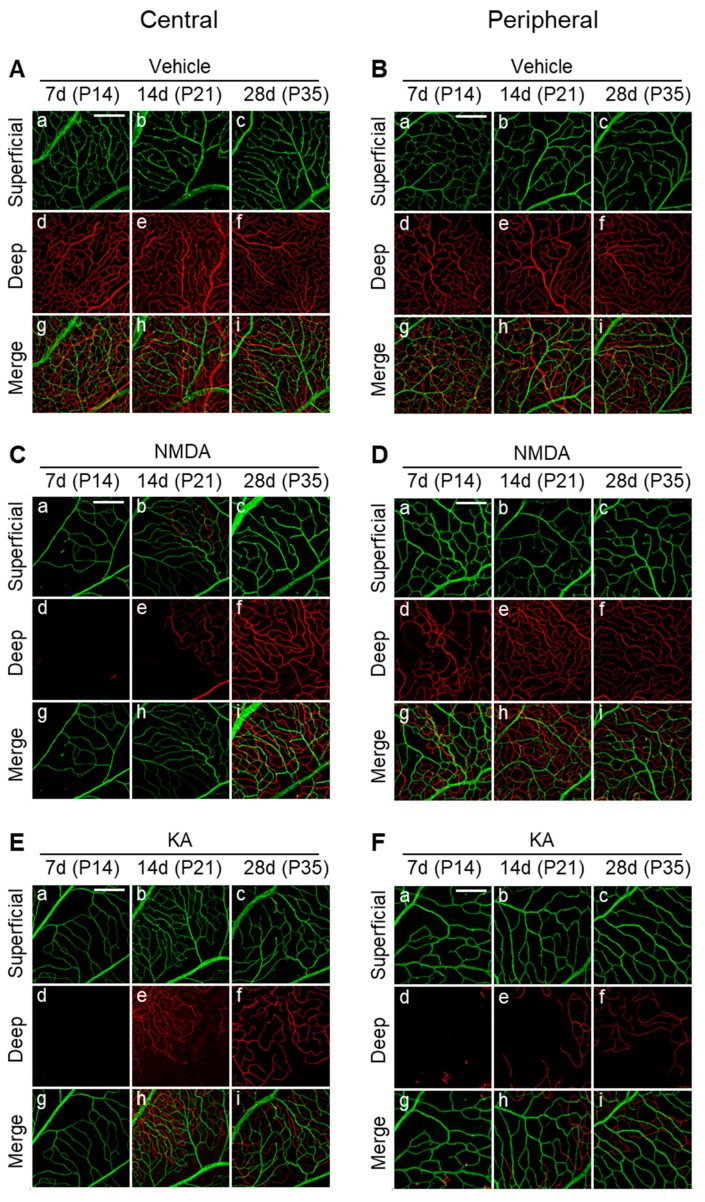
Formation of retinal superficial and deep vascular plexuses in rats intravitreally injected with vehicle, NMDA, or KA. Confocal microscopy images of retinal whole-mounts stained with an endothelial cell marker (RECA). Color depth projections show the vascular plexuses at the central and peripheral retinal areas as a single projection, in which distinct depth levels are color-coded. Superficial and deep blood vessels are indicated by green and red, respectively. (**A**,**B**) Vehicle; (**C**,**D**) NMDA; (**E**,**F**) KA. Scale bar = 150 µm in a (applies to b–i).

The development of superficial and deep vascular plexuses was examined by confocal microscopic imaging. Confocal images of the vascular network at the central retina were separated into the vascular plexuses at two different depth (superficial and deep) levels of the retina. In vehicle-treated eyes, superficial and deep vascular plexuses were formed in both the central and peripheral retinas (Figure 3A,B). In the NMDA-treated eyes, regression of superficial capillaries and delay in the formation of deep vascular plexus were observed seven days (on P14) after the injection. However, angiogenesis and re-vascularization occurred 14 days (on P21) after injection of NMDA, and superficial and deep vascular plexuses were formed 28 days (on P35) after the injection (Figure 3C,D). Similar observations were made in the KA-treated eyes (Figure 3E,F). The effects of NMDA on the vascular plexus in the central retina were more severe than in the peripheral retina (Figure 3C,D), but there was no apparent difference between the central and peripheral retinas in the KA-treated eyes (Figure 3E,F).

**Figure 4:**
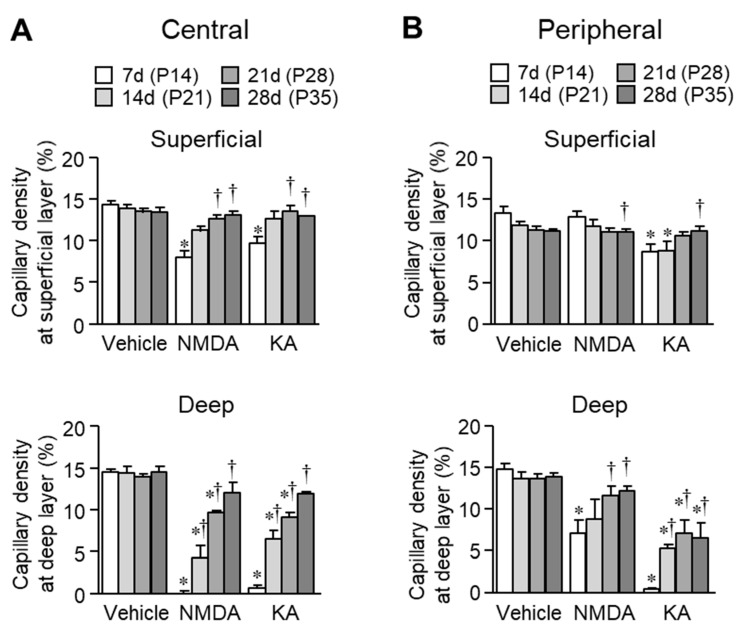
Quantification of area densities of the superficial and deep vascular plexuses in the central (**A**) and peripheral (**B**) retinal areas. * *p* < 0.05 vs. the corresponding age-matched control value (vehicle). ^†^ *p* < 0.05 vs. the corresponding seven days value after the injection. *n* = 4–5.

The quantitative data are summarized in Figure 4. In the central retina, capillary densities were significantly reduced at both the superficial and deep vascular plexuses seven days (on P14) after intravitreal injection of NMDA or KA, compared with the age-matched vehicle-treated eyes (Figure 4A). Thereafter, capillary densities increased in a time-dependent manner and reached normal levels 28 days (on P35) after the injection. In contrast, no reduction in capillary density at the superficial layer in the peripheral area was observed seven days (on P14) after the NMDA injection (Figure 4B). At the deep layer, capillary density decreased seven days (on P14) after intravitreal injection of NMDA or KA, but thereafter increased; however, the values did not reach the normal range. Thus, changes in vasculature were larger in the central retina than in the peripheral retina in NMDA-treated eyes. Such a region-difference was not observed in the retinas of KA-treated eyes.

**Figure 5:**
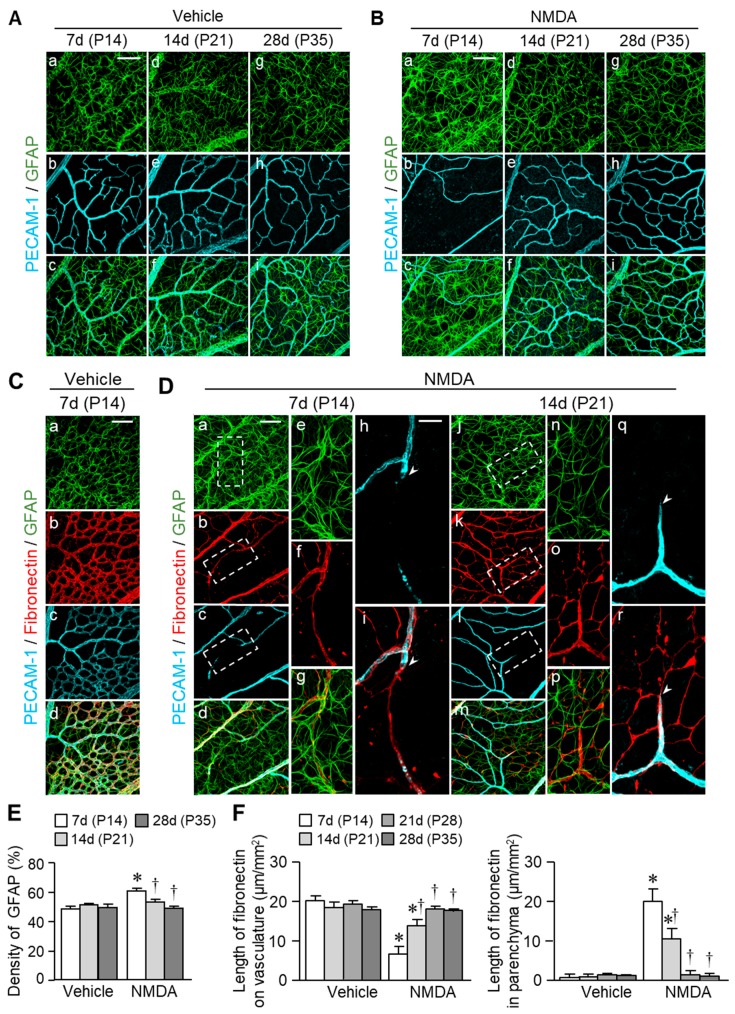
Changes in the distribution of astrocytes and fibronectin in the retinas of rats intravitreally injected with vehicle or NMDA. (**A**–**D**) Confocal microscopy images of retinal whole-mounts stained for astrocytes (GFAP), fibronectin, or endothelial cells (PECAM-1) 7 (P14), 14 (P21), and 28 days (P35) following intravitreal injection of vehicle (**A**,**C**) or NMDA (200 nmol; **B**,**D**). In panel D, higher-magnification images of the insets are shown in the panels De-i and Dn-r. Scale bars = 100 µm in Aa (applies to Ab–f), 100 µm in Ba (applies to Bb–i), 100 µm in Ca (applies to Cb–d), 100 µm in Da (applies to Db–d and Dj–m), and 30 µm in Dh (applies to Di, Dq, and Dr). (**E**,**F**) Quantification of the area density of GFAP (**E**), and the length of fibronectin on blood vessels and in retinal parenchyma in the central retinal area (**F**). * *p* < 0.05 vs. the corresponding age-matched control value (vehicle). ^†^ *p* < 0.05 vs. the corresponding 7 days value after the injection. *n* = 4–6.

Marked capillary regression was detected in the retinas of NMDA-treated eyes. Therefore, detailed and careful analyses of re-vascularization were performed in NMDA-treated eyes. We first examined the changes in the distribution of astrocytes, one of the retinal glial cells, because the glial cells play an important role in the development and maintenance of vasculature in the retina [21,27]. Double labeling with glial fibrillary acidic protein (GFAP; an astrocyte marker) and platelet endothelial cell adhesion molecule (PECAM-1; an endothelial cell marker) revealed that in the retinas of vehicle-treated eyes, GFAP-positive astrocytes had typical star shapes with several processes; some processes from astrocytes extended toward the vessels, and the end-feet of glial processes contacted and enveloped the vasculature (Figure 5A). Seven days after intravitreal injection of NMDA (on P14), morphological changes in astrocytes occurred and the area density of GFAP was increased (Figure 5Ba and E). These changes in astrocytes were later returned to almost normal levels (Figure 5Bd, Bg, and E).

Astrocytes produce fibronectin, which provides a template for the migration of endothelial cells [24,25,26]. We next investigated the changes in the distribution of fibronectin in the retinas of NMDA-treated eyes. In vehicle-treated eyes, fibronectin was found as a component of vascular basement membranes in the retinas (Figure 5C). Seven days (on P14) after intravitreal injection of NMDA, fibronectin was associated with astrocytes in retinal parenchyma (Figure 5Da–g) and found at vascular basement membranes and empty sleeves of basement membranes (Figure 5Dh and Di). Fibronectin in retinal parenchyma formed a network structure 14 days (on P21) after NMDA injection (Figure 5Dk), and the network pattern resembled that of astrocytes (Figure 5Dj, Dk, and Dn–p). Moreover, angiogenic sprouts from existing blood vessels were present along the fibronectin network (Figure 5Dq and Dr). No immunoreactivity of fibronectin was detected in retinal parenchyma 21 (on P28) and 28 days (on P35) after NMDA injection (data not shown). Seven days (on P14) after injection of NMDA, the length of fibronectin associated with blood vessels was shorter, but that in the retinal parenchyma was longer compared with the vehicle-treated eyes (Figure 5F). A similar relationship among fibronectin, astrocytes, and blood vessels was observed in the retinas of KA-treated eyes (Supplementary Figure S1). Therefore, the pattern and timing of fibronectin network formation is likely to affect re-vascularization in retina with neurodegenerative injuries.

**Figure 6:**
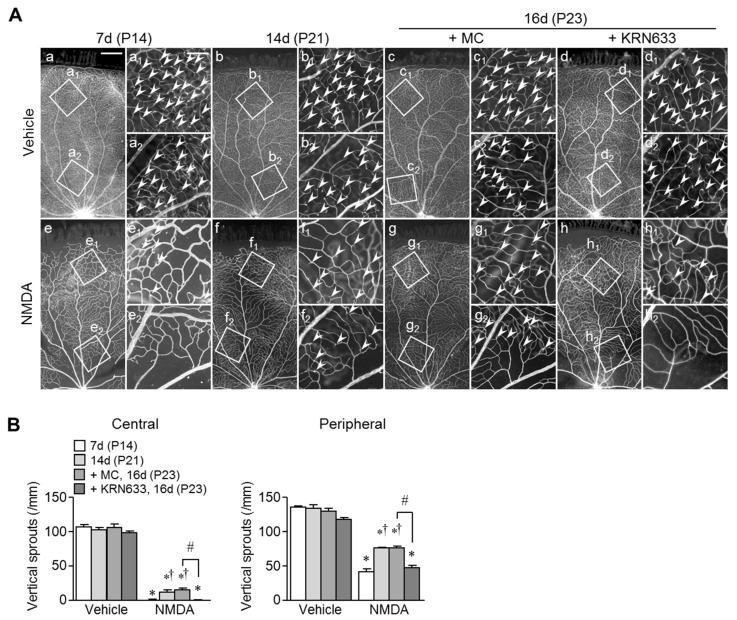
Effects of pharmacological blockade of VEGF signaling pathway on angiogenesis and re-vascularization in retinas of rats intravitreally injected with vehicle or NMDA. (**A**) Fluorescence photomicrographs of retinal whole-mounts stained for endothelial cells (RECA) obtained 7 (P14), 14 (P21), and 16 days (P23) following intravitreal injection of vehicle or NMDA (200 nmol) on P7. The animals were injected with KRN633 or the vehicle (0.5% methylcellulose; MC) on P21 and P22. Higher-magnification images of the insets are shown in the panels Aa_1_–h_1_ and Aa_2_–h_2_. Arrowheads indicate vertical sprouts. Scale bars = 500 µm in Aa (applies to b–h) and 150 µm in Aa_1_ (applies to b_1_–h_1_ and a_2_–h_2_). (**B**) Quantification of the number of vertical sprouts in central and peripheral retinal areas. * *p* < 0.05 vs. the corresponding age-matched control value (vehicle or vehicle + MC). ^†^ *p* < 0.05 vs. the corresponding seven days value after the injection. ^#^ *p* < 0.05. *n* = 4.

To determine the roles of VEGF in re-vascularization in the retinas of NMDA-treated eyes, we examined the effects of a two-day treatment with KRN633, an inhibitor of VEGF receptor tyrosine kinase [28], or the vehicle on retinal blood vessels at P21 and P22 (i.e., 14 and 15 days after intravitreal injection). Treatment with KRN633 had no significant effect on retinal vasculature in vehicle-treated eyes (Figure 6Aa–d and B). Re-vascularization occurred and increased vertical sprouts were observed in the central and peripheral retinas of NMDA-treated eyes, but the responses were completely suppressed by treatment with KRN633 (Figure 6Ae–h and B). These results suggest that re-vascularization in the retinas of NMDA-treated eyes occurs in a VEGF-dependent manner.

KRN633 did not affect the superficial and deep blood vessels in retinas of vehicle-treated eyes (Supplementary Figure S2A, C, and D), whereas it suppressed the increase in capillary densities at both superficial and deep vascular plexuses in the central retina of NMDA-treated eyes (Supplementary Figure S2B–D). The deep vascular plexus in NMDA-treated eyes is highly sensitive to the VEGF inhibitor, suggesting that the population of immature blood vessels in the deep plexus is higher than that in the superficial plexus, and most capillaries in the deep plexus exhibit VEGF dependency at this time point.

**Figure 7:**
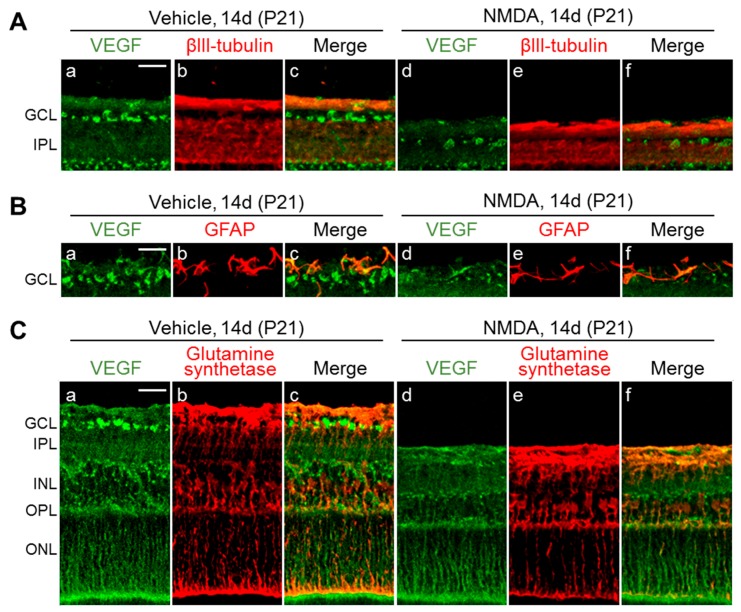
Colocalization of VEGF and cell type-specific markers in retinas of rats intravitreally injected with vehicle or NMDA. Cryosections stained with anti-VEGF antibody (green) plus one of the indicated cell-specific antibodies (red) obtained 14 days (P21) following intravitreal injection of vehicle or NMDA. (**A**) βIII-tubulin; (**B**) GFAP; (**C**) Glutamine synthetase. Scale bar = 150 µm in a (applies to b–f).

We also examined the distribution of VEGF and VEGF expressing cells 14 days (on P21) after NMDA injection. In vehicle-treated eyes, immunoreactivities for VEGF were detected in cells marked with GFAP and βIII tubulin (a marker for astrocytes and RGCs, respectively) in GCL and IPL. Horizontally oriented processes with VEGF immunoreactivity in IPL and OPL were labeled with antibodies for GS, a marker for Müller cells. The staining pattern of VEGF was almost the same between the vehicle- and NMDA-treated eyes. However, a difference in the thickness of the inner retinal layer was observed (Figure 7).

## 3. Discussion

The present study demonstrates that suppression of vascular growth and regression of capillaries occur in the neonatal rat retina following intravitreal injection of NMDA or KA. However, re-vascularization occurs several days after the intravitreal injection in a VEGF-dependent manner. In NMDA- and KA-induced retinal injury model, fibronectin produced by astrocytes acts as a scaffold for angiogenesis from existing blood vessels. In normal retinas, neuronal cells and glial cells are the main sources of VEGF, whereas in the injured retina, glial cells may be the predominant source of VEGF. These results suggest that glial cells play an important role in angiogenesis in neonatal rat models of retinal neurodegeneration.

Interruption of retinal vascular development was commonly observed in the NMDA- and KA-induced retinal injury models. However, the degree of the interruption in the central retina was larger than that in the peripheral retina of NMDA-treated eyes. On the other hand, such region-difference was not observed in the KA-treated eyes. The degree of vascular development interruption seems to be correlated with the degree of neuronal injury [19]. Indeed, NMDA-induced neuronal injury showed a tendency to be more severe in the central retina than in the peripheral retina, whereas this tendency was not observed in KA-induced neuronal injury. The region-dependent susceptibility of retinal cells to neurotoxic agents may be explained by the region-dependent expression levels of receptors for NMDA and KA [29,30,31,32,33].

Following a brief period of vascular development interruption, endothelial cell re-growth occurred in the retinas of NMDA- and KA-treated eyes. The key contributor for angiogenesis could be hypoxia, which leads to an enhancement of VEGF production [27,34]. In this study, we found that pharmacological blockade of the VEGFR signaling pathway with KRN633 completely suppressed angiogenesis in the retinas of NMDA-treated eyes. However, interestingly, no enhancement of VEGF expression was observed in NMDA-treated eyes. In NMDA-treated eyes, the number of retinal cells decreased. Therefore, the remaining capillaries may provide sufficient blood flow to survived retinal cells. As a result, severe retinal hypoxia and enhancement of VEGF expression did not occur even after the regression of retinal capillaries in NMDA-treated eyes. Thus, no aggressive angiogenesis and formation of abnormal blood vessels with irregular diameter and misdirection were observed in the injured retina. This observation was consistent with our previous study, demonstrating that NMDA-induced loss of the inner retinal neurons completely prevented the formation of retinopathy of prematurity-like pathological retinal blood vessels [35].

A scaffold for endothelial cell migration is also important for angiogenesis. It has been known that fibronectin produced by astrocytes acts as a scaffold for angiogenesis from existing blood vessels [24,26]. Consistent with the results of previous studies [5,7,24,26], astrocytes were activated immediately following NMDA-induced retinal injury, as indicated by the increased area density of GFAP. In addition, our immunohistochemical data revealed that fibronectin increased in retinal parenchyma after NMDA injection and that the source of fibronectin is activated astrocytes. Unlike immature astrocytes, mature normal astrocytes do not produce fibronectin [24]. Our results support this concept because fibronectins were present as a component of vascular basement membranes, but not in parenchyma, in the P14 control retinas. In contrast, activated astrocytes have the potential to produce fibronectin and form the fibronectin network. Angiogenic sprouts from existing blood vessels were associated with the fibronectin network in retinal parenchyma. These results suggest that fibronectin secreted from activated astrocytes serves as a scaffold for re-vascularization in neonatal rat retina with neurodegenerative injuries.

In the present study, we used neonatal rats for two reasons: (1) developing retinal blood vessels are more sensitive to neuronal cell degeneration than established retinal vasculature [36] and (2) higher levels of VEGF are present in neonatal retinas and the situation facilitates VEGF-dependent angiogenesis. Needless to say, most retinal neurovascular degenerative diseases occur in middle-aged and older adults. Therefore, further study will be needed to determine how glial cells contribute to angiogenesis in adult rat retina with neurodegenerative injuries.

In summary, the present study provides the first evidence that angiogenesis and re-vascularization occur following capillary regression in the neonatal retina with neurodegenerative injury. The results of mechanistic analyses suggest that astrocytes activated by the injury produce and secrete fibronectin to form a scaffold for endothelial cell migration. VEGF released mainly from glial cells stimulates the process of angiogenesis. Thus, neonatal rats intravitreally injected with NMDA or KA could serve as an animal model for studying the mechanisms underlying capillary regression and angiogenesis/revascularization under retinal neurovascular degenerative diseases such as diabetic retinopathy and glaucoma.

## 4. Materials and Methods

### 4.1. Animals

All animal procedures were approved by the Institutional Animal Care and Use Committee at Kitasato University (Approval Number: 17-27, Approved on 8 December 2017) and handled in accordance with the Association for Vision Research and Ophthalmology (ARVO) Statement for the Use of Animals in Ophthalmic and Vision Research.

Pregnant Sprague–Dawley rats were obtained from Charles River Breeding Laboratories (Tokyo, Japan) and maintained under a 12 h light–dark cycle with free access to food and water. The day of birth was noted by daily inspections and defined as postnatal day (P)0.

### 4.2. Retinal Neurodegenerative Injury Models

Seven-day-old rats were intravitreally injected with 200 nmol of NMDA (Nacalai Tesque, Kyoto, Japan) or 20 nmol of KA (Tocris Bioscience, Ellisville, MO, USA) according to methods described previously [19,36]. Briefly, under general anesthesia, the eye was opened by cutting along the fused junctional epithelium where the two eyelids come together, and NMDA or KA in a total volume of 2 μL was injected into the vitreous cavity of one eye. The same volume of vehicle (saline) was injected into the vitreous cavity of the other eye as a control.

### 4.3. Histological Assessment of The Retina

To investigate retinal morphometric changes, retinal cross-sections stained with hematoxylin and eosin were used [19,36]. In brief, rats were sacrificed 7 (on P14, NMDA, *n* = 5; KA, *n* = 5), 14 (on P21, NMDA, *n* = 5; KA, *n* = 4), and 28 (on P35, NMDA, *n* = 5; KA, *n* = 4) days after intravitreal injection of NMDA or KA, and their eyes were enucleated. The eyes were fixed in Davidson’s solution (37.5% ethanol, 9.3% formaldehyde, 12.5% acetic acid, and 3% glutaraldehyde) for 12 h. Fixed eyes were embedded in paraffin. Cross-sections (5 μm) were cut through the optic disc of the eye and were examined between 500 and 750 μm from the optic nerve head or the peripheral edge of retina. The number of cells in the ganglion cell layer (GCL) was counted and the thicknesses of the inner plexiform layer (IPL) and the inner nuclear layer (INL) were measured. The values were averaged for each eye.

### 4.4. Immunohistochemistry

Immunohistochemistry was performed, according to methods reported previously [19,37]. In brief, rats were deeply anesthetized with pentobarbital sodium, followed by systemic perfusion via the aorta with 1% paraformaldehyde in phosphate-buffered saline (PBS) (pH 7.4). After perfusion, eyes were removed and processed for whole-mount preparations or cross-sectional preparations. For whole-mount preparations, eyes were stored in a fixative agent for 24 h at 4 °C, and the retina was separated from the lens, vitreous, and pigment epithelium. For cross-sectional preparations, eyes were stored in fixative for 1 h at 4 °C, then rinsed several times with PBS, infiltrated overnight with 30% sucrose in PBS at 4 °C, and frozen in optimum cutting temperature (OCT) compound (Sakura Finetek, Torrance, CA, USA).

### 4.5. Assessment of Retinal Vasculature

Eyes were obtained 7 (on P14, NMDA, *n* = 4; KA, *n* = 4), 14 (on P21, NMDA, *n* = 4; KA, *n* = 4), 21 (on P28, NMDA, *n* = 5; KA, *n* = 4), and 28 (on P35, NMDA, *n* = 5; KA, *n* = 4) days after intravitreal injection of NMDA or KA. Retinas were stained with an endothelial cell marker, according to similar methods reported previously [19,36]. In brief, retinas were incubated for 0.5–1 h in blocking solution (5% normal hamster serum) in PBS, containing 0.3% Triton X-100 (PBS-T). Retinas were then incubated for 12–15 h with a mouse monoclonal anti-rat endothelial cell antigen (RECA)-1 antibody (endothelial cell marker) (1:800; Bio-Rad, Hercules, CA, USA), followed by incubation with a Cy3-conjugated donkey antibody against mouse immunoglobulins (1:400; Jackson ImmunoResearch Laboratories, West Grove, PA, USA). Tissues were rinsed in PBS-T and retinal whole-mounts were prepared using Vectashield mounting media (Vector Laboratories, Burlingame, CA, USA).

The size of the avascular area and the number of vertical sprouts were measured using images obtained by a BZ-9000 fluorescent microscope system (Keyence, Osaka, Japan).

The development of superficial and deep vascular plexuses was examined, according to methods previously described [38]. In brief, a series of confocal optical sections (20× objective) of the central and the peripheral areas in whole-mounts were taken by a confocal laser scanning microscope LSM 710 (Zeiss, Oberkochen, Germany). The series began at the surface of the retina and extended through the GCL into the OPL, at different focal planes using a 2-μm-step (z-series). Z-projections of confocal stacks for superficial or deep vascular plexuses were generated using a color-coding method in ZEN 2010 imaging software (Zeiss).

### 4.6. Changes in Astrocytes and Fibronectins

In the neonatal rat retina, astrocytes produce fibronectin and provide a template for migration of endothelial cells [24,26]. Therefore, we examined the relationship among astrocytes, fibronectin, and blood vessels. In these experiments, retinal whole-mounts were labeled with a Cy3-conjugated mouse monoclonal anti-GFAP antibody (1:500, Sigma-Aldrich, St. Louis, MO, USA) for astrocytes, a rabbit polyclonal anti-fibronectin antibody (1:500, Dako, Glostrup, Denmark) for fibronectin, and a goat polyclonal anti-PECAM-1 antibody (1:500; R&D Systems, Minneapolis, MN, USA) for endothelial cells.

We evaluated the morphological changes of astrocytes and the distribution of fibronectin in the central retinal area 7 (on P14, *n* = 12), 14 (on P21, *n* = 12), 21 (on P28, *n* = 6), and 28 (on P35, *n* = 10) days after intravitreal injection of saline or NMDA. In some experiments, we examined the morphological changes of astrocytes and the distribution of fibronectin 7 and 14 days after intravitreal injection of KA. The morphological changes of astrocytes were assessed by measuring area density of GFAP. The length of fibronectin network associated with blood vessels and presented in retinal parenchyma in 3 fields of central retinal area was measured (Supplementary Figure S3). The values were averaged for each retina.

### 4.7. Role of VEGF in Re-Vascularization in Retinal Neurodegenerative Injury Models

To examine the roles of VEGF in re-vascularization in retinal neurodegenerative injury models, neonatal rats intravitreally injected with saline or NMDA (200 nmol/eye) on P7 were treated subcutaneously with KRN633 (10 mg/kg/day, *n* = 4), a small-molecule inhibitor of VEGF receptor tyrosine kinase, or vehicle (0.5% methylcellulose, *n* = 4) 14 and 15 days after intravitreal injection (i.e., on P21 and P22) and sacrificed the next day (i.e., 16 days after intravitreal injection). For comparison, we examined the vasculature 7 (on P14, *n* = 4) and 14 (on P21, *n* = 4) days after the intravitreal injection. The dose of KRN633 was selected based on the results of our previous studies [19,35,39]. KRN633 was synthesized in-house at Kitasato University (Dr. Tohru Nagamitsu, Department of Organic Synthesis).

### 4.8. Distribution of VEGF in The Retina

Retinal cross-sections stained with anti-VEGF antibodies were used, according to similar methods reported previously [19]. The 16-μm-thick sections were rinsed to remove the OCT compound, incubated in blocking solution in PBS-T for 0.5–1 h, and then incubated for 12–15 h with primary antibodies.

To visualize the distribution of immunoreactivity for VEGF and VEGF expressing cells, the following primary antibodies were used: a goat polyclonal anti-VEGF antibody (1:500; R&D Systems) and a Cy3-conjugated GFAP antibody for astrocytes, a mouse monoclonal anti-βIII tubulin antibody (1:2000; Promega, Madison, WI, USA) for RGCs, or a mouse monoclonal anti-glutamine synthetase (GS) antibody (1:1000; EMD Millipore, Billerica, MA, USA) for Müller cells. After incubation with primary antibodies, the sections were rinsed several times with PBS-T and incubated for 4 h with fluorescently labeled, species-specific secondary antibodies (1:400; Jackson ImmunoResearch Laboratories). The sections were rinsed in PBS-T and mounted with Vectashield containing 4’,6-diamidino-2-phenylindole (Vector Laboratories).

### 4.9. Data Analysis

Data are expressed as means ± SE. The significance of the differences between the mean values was evaluated with ANOVA followed by Tukey’s test for multiple comparisons (GraphPad, San Diego, CA, USA). *p*-values of less than 0.05 were regarded as statistically significant.

## Supplementary Materials

Supplementary materials can be found at https://www.mdpi.com/1422-0067/20/19/4759/s1, Figure S1: Changes in distribution of astrocytes and fibronectin in retinas of rats intravitreally injected with KA. Confocal microscopy images of retinal whole-mounts stained for astrocytes (GFAP), fibronectin, orendothelial cells (PECAM-1) 7 (P14) and 14days (P21) following intravitreal injection of KA (20 nmol). Higher-magnification images of the inset are shown in the panels E–I and N–R. Scalebars = 100 μm in A (applies to B–D and J–M) and 30 μm in H (appliestoI, Q, and R); Figure S2: Effects of pharmacological blockade of VEGF signaling pathway on the formation of superficial and deep vascular plexuses in retinas of rats intravitreally injected with vehicleor NMDA. (A,B) Confocal microscopy images of retinal whole-mounts stained for endothelial cells (RECA) obtained 7 (P14), 14 (P21), and 16 days (P23) following the injection. The animals were treated with KRN633 or the vehicle (0.5%methylcellulose;MC) on P21 and P22. Colour depth projections show the vascular beds at central and peripheral areas as a single projection, in which distinct depth levels are color-coded. The superficial and deep vascular plexuses are indicated by green and red, respectively. Scalebar = 150 μm in a (applies to b-h). (C,D) Quantification of capillary area density of each plexusin central and peripheral retinalareas.* *P* < 0.05 vs. the corresponding age-matched control value (vehicle). ^†^ < 0.05 vs. the corresponding 7 days value after the injection. ^#^ *P* < 0.05. *n* = 4; Figure S3: The method for analyses of the network of fibronectin in retinal flat-mount preparations. Fibronectin associated with capillaries (B,upper) and fibronectin in retinal parenchyma (B,lower) were traced in confocal microscopy images of retinal whole-mounts labeled with anti-fibronectin antibodies and anti-PECAM-1 antibodies.The sum of length of traced fibronectin was calculated. Scalebar = 150 μm in Aa.
