# Supplementary material for: Cellular Mechanisms of Angiogenesis in Neonatal Rat Models of Retinal Neurodegeneration
Source: Int J Mol Sci. 2019 Sep 25;20(19):4759. doi: 10.3390/ijms20194759 (PMC6801463; doi:10.3390/ijms20194759)
Supplement: Supplementary file 1 [file ijms-20-04759-s001.pdf]

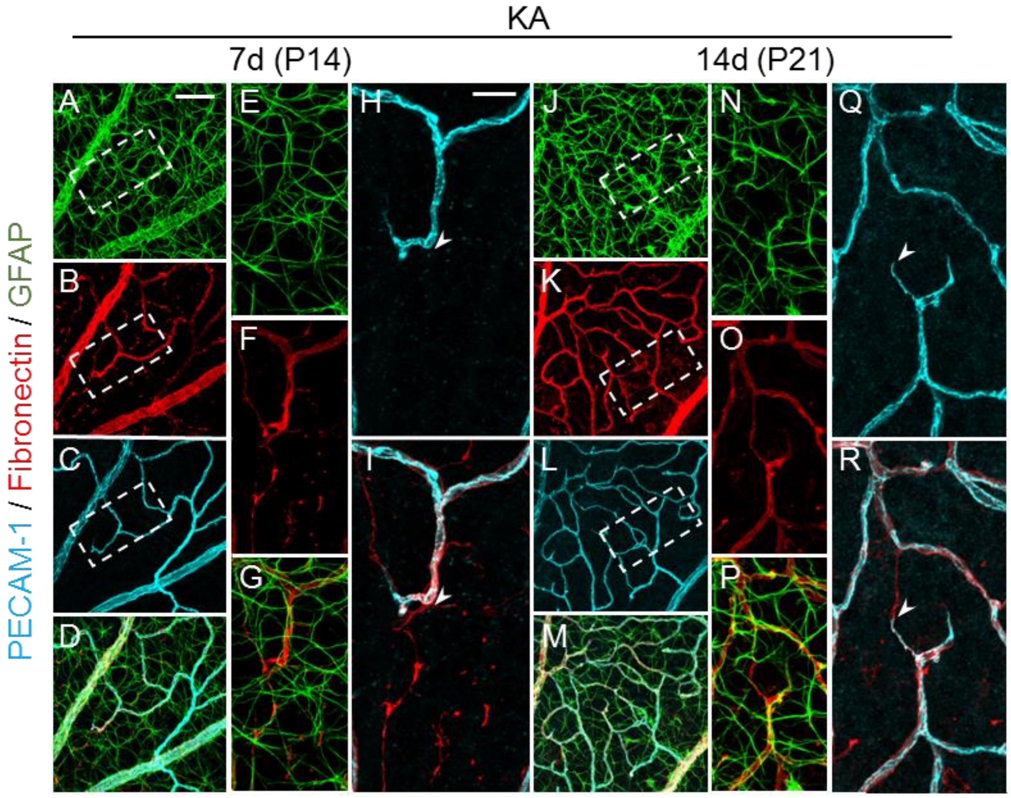

Supplementary Figure 1: Changes in distribution of astrocytes and fibronectin in retinas of rats intravitreally injected with KA. Confocal microscopy images of retinal whole-mounts stained for astrocytes (GFAP), fibronectin, or endothelial cells (PECAM-1) 7 (P14) and 14 days (P21) following intravitreal injection of KA (20 nmol). Higher-magnification images of the inset are shown in the panels E-I and N-R. Scale bars = 100 μm in A (applies to B-D and J-M) and 30 μm in H (applies to I, Q, and R).

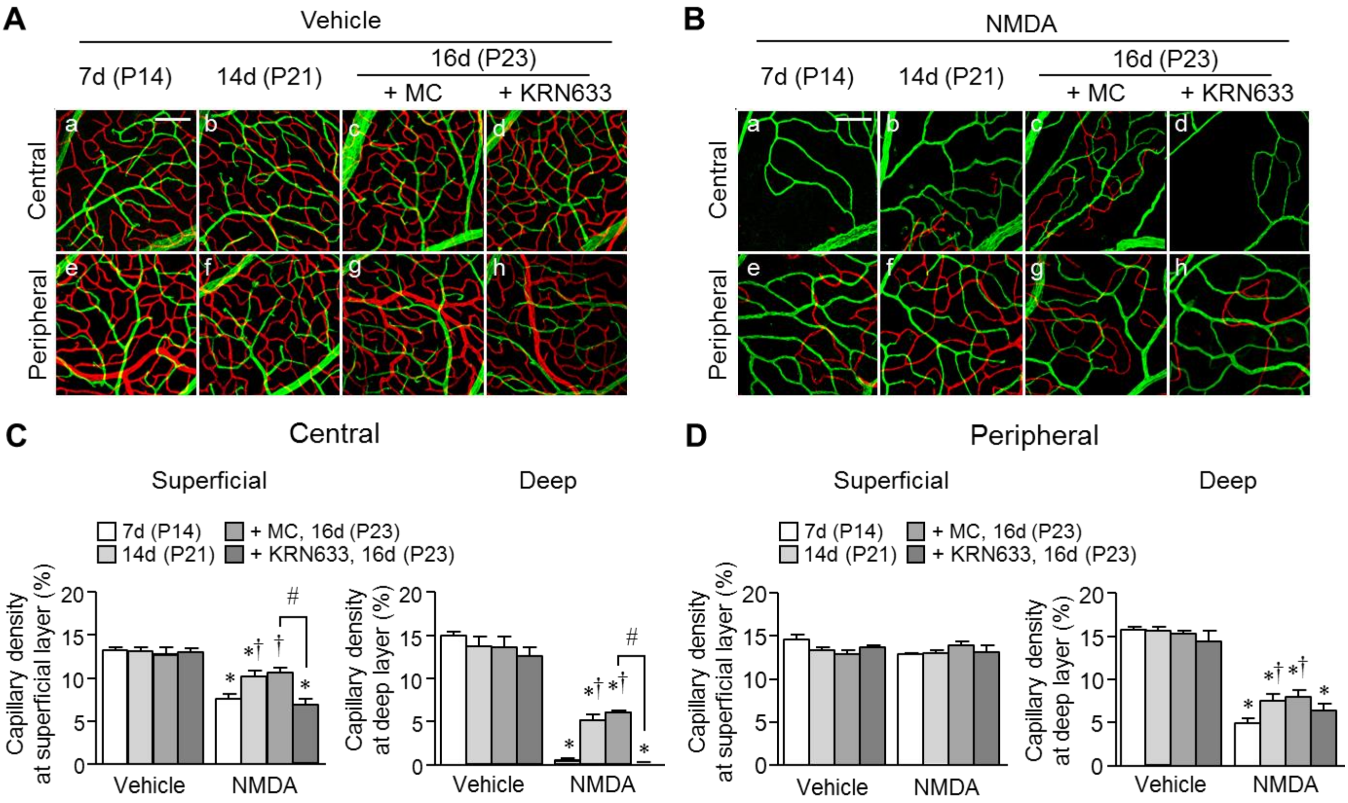

Supplementary Figure 2: Effects of pharmacological blockade of VEGF signaling pathway on the formation of superficial and deep vascular plexuses in retinas of rats intravitreally injected with vehicle or NMDA. (A, B) Confocal microscopy images of retinal whole-mounts stained for endothelial cells (RECA) obtained 7 (P14), 14 (P21), and 16 days (P23) following the injection. The animals were treated with KRN633 or the vehicle (0.5% methylcellulose; MC) on P21 and P22. Colour depth projections show the vascular beds at central and peripheral areas as a single projection, in which distinct depth levels are color-coded. The superficial and deep vascular plexuses are indicated by green and red, respectively. Scale bar = 150 μm in a (applies to b-h). (C, D) Quantification of capillary area density of each plexus in central and peripheral retinal areas. *$P < 0.05$ vs. the corresponding age-matched control value (vehicle). †$P < 0.05$ vs. the corresponding 7 days value after the injection. #$P < 0.05$. n = 4

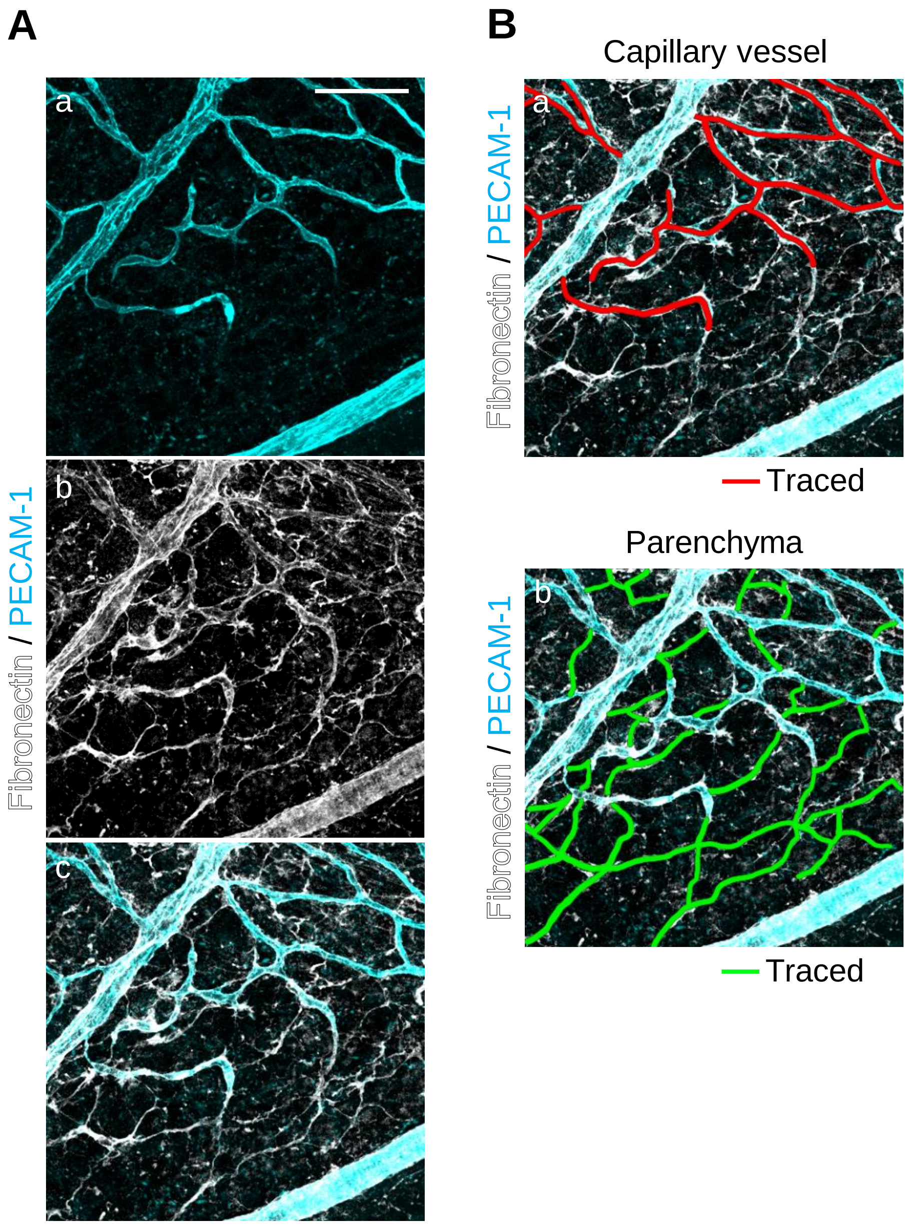

Supplementary Figure 3: The method for analyses of the network of fibronectin in retinal flat-mount preparations. Fibronectin associated with capillaries (B, upper) and fibronectin in retinal parenchyma (B, lower) were traced in confocal microscopy images of retinal whole-mounts labeled with anti-fibronectin antibodies and anti-PECAM-1 antibodies. The sum of length of traced fibronectin was calculated. Scale bar = 150 μm in Aa.
